# Genomic population structure of freshwater‐resident and anadromous ide (*Leuciscus idus*) in north‐western Europe

**DOI:** 10.1002/ece3.1909

**Published:** 2016-01-22

**Authors:** Mikkel Skovrind, Morten Tange Olsen, Filipe Garrett Vieira, George Pacheco, Henrik Carl, M. Thomas P. Gilbert, Peter Rask Møller

**Affiliations:** ^1^ Section for Evolutionary GenomicsNatural History Museum of DenmarkUniversity of CopenhagenØster Voldgade 5‐71350Copenhagen KDenmark

**Keywords:** Anadromous, genomic population structure, Genotyping‐by‐Sequencing, *Leuciscus idus*, salinity, teleost

## Abstract

Climate change experts largely agree that future climate change and associated rises in oceanic water levels over the upcoming decades, will affect marine salinity levels. The subsequent effects on fish communities in estuarine ecosystems however, are less clear. One species that is likely to become increasingly affected by changes in salinity is the ide (*Leuciscus idus*). The ide is a stenohaline freshwater fish that primarily inhabits rivers, with frequent anadromous behavior when sea salinity does not exceed 15%. Unlike most other anadromous Baltic Sea fish species, the ide has yet to be subjected to large‐scale stocking programs, and thus provides an excellent opportunity for studying the natural population structure across the current salinity gradient in the Danish Belts. To explore this, we used Genotyping‐by‐Sequencing to determine genomic population structure of both freshwater resident and anadromous ide populations in the western Baltic Sea region, and relate the results to the current salinity gradient and the demographic history of ide in the region. The sample sites separate into four clusters, with all anadromous populations in one cluster and the freshwater resident populations in the remaining three. Results demonstrate high level of differentiation between sites hosting freshwater resident populations, but little differentiation among anadromous populations. Thus ide exhibit the genomic population structure of both a typical freshwater species, and a typical anadromous species. In addition to providing a first insight into the population structure of north‐western European ide, our data also (1) provide indications of a single illegal introduction by man; (2) suggest limited genetic effects of heavy pollution in the past; and (3) indicate possible historical anadromous behavior in a now isolated freshwater population.

## Introduction

It is widely accepted that oceanic water levels will rise and lead to changes in salinity as an implication of climate change (ICCP 2014). In some regions salinity will increase (areas with decreased precipitation), while other areas will see decreases (areas with increased precipitation) (Durack and Wijffels 2010). The Baltic Sea is a mega estuary with salinities ranging from 0 to 20% (Janssen et al. 1999) in which the salinity is predicted to decline throughout the 21st century (Neumann 2010; Meier et al. 2012), leading to changes in species distributions (Vuorinen et al. 2015). However, no published models for salinity in the Baltic Sea incorporate the impact of rising sea levels, making any predictions of future salinities uncertain (Andersson et al. 2015). Nevertheless, any changes will have great impact on anadromous populations of any freshwater fish species that spend part of their lifecycles in the brackish Baltic Sea (e.g., pike *Esox lucius*, perch *Perca fluviatilis*, roach *Rutilus rutilus*, and ide *Leuciscus idus*) (Müller and Berg 1982; Engstedt et al. 2010; Skovrind et al. 2013; Rohtla et al. 2015). Currently, in the western part of the Baltic Sea, species such as these migrate into brackish water close to their maximum salinity tolerance. Such migrations are different to those of species such as salmon, as they do not undergo a process of physiological adaptation to sea water (smoltification), but remain freshwater‐adapted throughout their life (Marchall and Grossel 2005). This limits anadromous behaviour of stenohaline freshwater fish to regions within their salinity tolerance, thus future salinity changes will affect the areas in which this anadromous behavior is possible, unless species can adapt to different salinity tolerances. This in turn will impact the connectivity between populations, and the overall population structure of such species.

**Figure 1:**
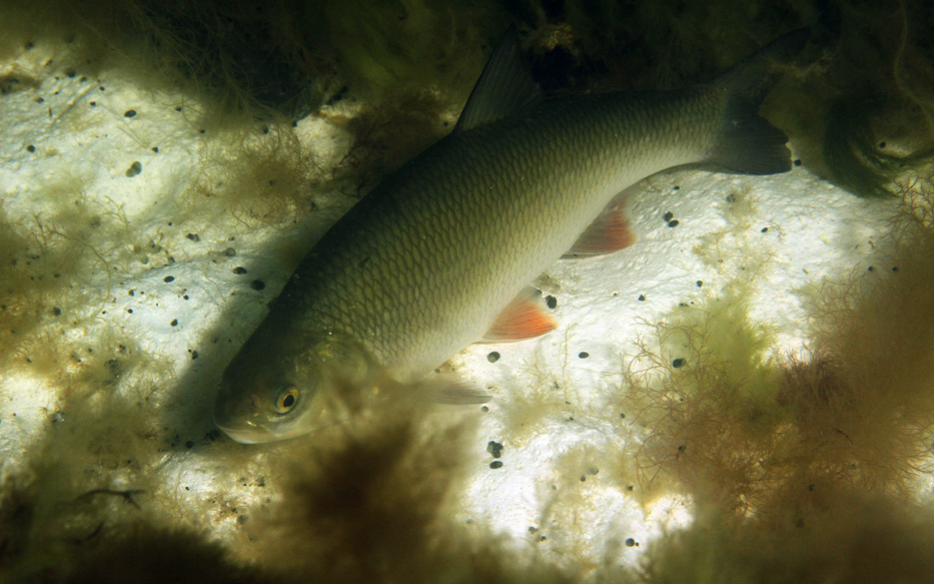
Photo of ide (*Leuciscus idus*) from the brackish coastal waters of Stevns Klint, south‐eastern Denmark, September 2014.

The maximum tolerated salinity for ide (Fig. 1) is 15% (Penthon 1985; van Beek 1999). Thus anadromous ide populations that today live in the transition zone between the brackish Baltic Sea and the marine North Sea are believed to be living on the edge of their physiological capacity. Clear evidence in support of this comes from observations that influxes of higher salinity oceanic water from the North Sea often kills thousands of anadromous individuals along the east coast of Zealand, Denmark (Carl 2012a). These observations also suggest that ide in this region may be under selection for adaptation to higher salinity tolerances.

Several previous studies have investigated the genetic structure of populations of other anadromous, stenohaline freshwater fishes, including perch *Perca fluviatilis*, pike *Esox lucius* and zander *Sander lucioperca*. Using D‐loop mitochondrial markers and microsatellites, these studies indicated genetic differentiation between coastal, anadromous and freshwater populations (Nesbö et al. 1998; Larsen et al. 2005; Säisä et al. 2010). Similar research on ide has been extremely limited to a small number of studies which used allozymes and focused on river stretches far from the sea (Wolter et al. 2003; Zhigileva et al. 2010, 2013). Furthermore, unlike most other anadromous fish species in the Baltic, the ide has not been subject to large‐scale stocking programmes, and it thus provides an excellent opportunity for studying the natural population structure of an anadromous fish species across a salinity gradient ranging from optimal, suboptimal to lethal habitats.

Given this, we applied a population genomic approach to identify genetic inter‐ and intravariability of ide populations in the Baltic Sea‐North Sea transition zone in north‐western Europe. In particular our analyses focused on the genomic relationship between freshwater residents and anadromous populations, in order to obtain a glimpse into what the future may hold for anadromous freshwater fishes as climate change transforms the salinities of their habitats.

## Methods

### Sampling and storage

**Figure 2:**
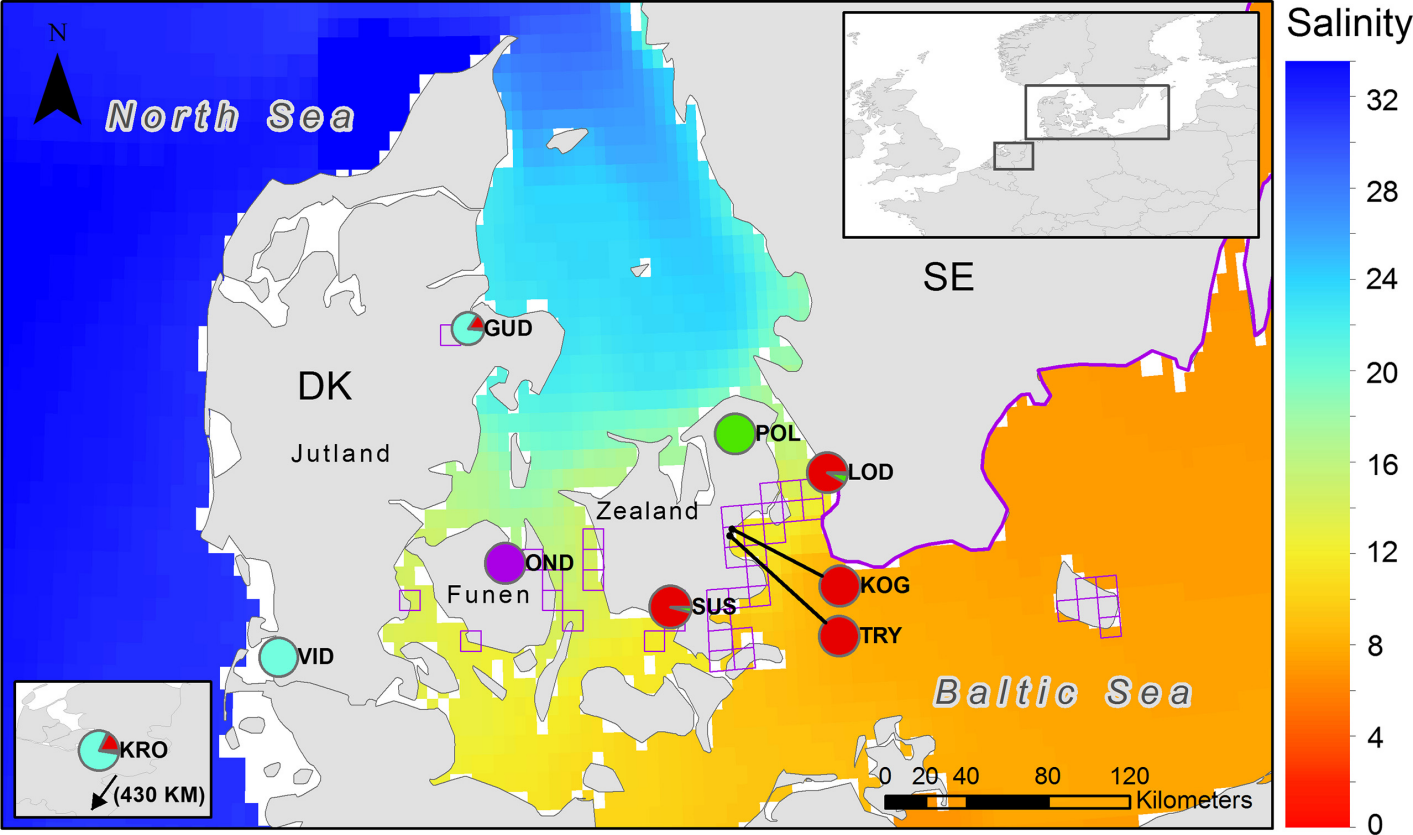
Geographic map of sample sites of ide included in this study. Pie colors indicate the summed ancestral fractions for each sample site for *K* = 4. Pie sizes are related to sample sizes. Purple squares indicate 10 × 10 KM quadrats in which ide have been registered in Danish marine waters since 1995. The presence of ide on the coast of Sweden is marked by a purple line (Kullander et al. 2012). Baltic Sea salinity data are mean salinity for the period 1999–2009. North Sea salinity data are mean salinity for the period 2007–2008.

Fin‐clips from 95 ide were collected from nine localities in Denmark, Sweden and the Netherlands by anglers and scientists (Table 1, Fig. 2). Samples were taken from live fish that were released alive immediately after sampling, except for a few voucher specimens now stored in the collection of the Natural History Museum of Denmark (Sample ID: ZMUC P265401‐02, P265454, and P265897‐5988). All samples were stored in 96% ethanol and kept in minus 20°C freezers. The sample sites fall into three categories: (1) Streams running into brackish water with salinities <15‰. (Susåen (SUS), Tryggevælde Å (TRY), Køge Å (KOG) and Lödde Ä (LOD)); (2) Streams running into marine water with salinities >15‰ (Kromme Rijn (KRO), Vidåen (VID), Gudenåen (GUD), Odense Å (GUD) and Odense Å (OND)); and (3) a stream, Pøle Å (POL), running into a freshwater lake that until the 17th century was a marine fjord (any migration to the sea from this lake is today hindered by a physical barrier). Samples were classified as anadromous when taken from streams in category one. In these streams large shoals of migratory ide are annually recorded by the authors or local anglers in the deltas, and the salinity outside the deltas is normally within the tolerated level. Freshwater resident status was given to samples from category two and three.

**Table 1:** Sample sites of ide in north‐western Europe

Site	Code	Country	Anadromous	*N*	Year	Num.	Eff Num	H_O_	H_S_	H_T_	G_IS_	G_IS_ P
Kromme Rijn	KRO	NL	No	11	2014	1.764	1.372	0.230	0.238	–	0.031	0.000
Vidåen	VID	DK	No	10	2013	1.581	1.309	0.197	0.193	–	−0.016	1.000
Gudenåen	GUD	DK	No	7	2013–14	1.640	1.357	0.238	0.228	–	−0.042	0.928
Odense Å	OND	DK	No	11	2014	1.630	1.337	0.208	0.210	–	0.009	0.000
Pøle Å	POL	DK	No	11	2014	1.603	1.327	0.210	0.203	–	−0.037	0.980
Susåen	SUS	DK	Yes	12	2013	1.777	1.387	0.237	0.245	–	0.033	0.000
Tryggevælde Å	TRY	DK	Yes	11	2009	1.804	1.398	0.259	0.254	–	−0.023	0.661
Køge Å	KOG	DK	Yes	11	2012	1.806	1.391	0.248	0.251	–	0.012	0.000
Lödde Ä	LOD	SE	Yes	11	2014	1.788	1.393	0.248	0.250	–	0.007	0.000
Total				95		2.000	1.319	0.231	0.230	0.264	−0.002	1.000

Site: Name of sample site. Code: Three letter code for sample sites. Country: National codes. Anadromous: Presence or Absence of anadromous behaviour. N: Number of individuals sampled. Num: Number of alleles. Eff Num: Effective number of alleles. H_O_: Observed heterozygosity. H_S_: Expected heterozygosity in populations. H_T_: Expected total heterozygosity. G_IS_: Inbreeding coefficient. G_IS_ *P*: Inbreeding coefficient *P* value. DK: Denmark; NL: Nederland; SE: Sweden.

### DNA extraction, quality‐, and quantity control

DNA was extracted using Qiagen's Blood and Tissue kit (Qiagen Ltd., Crawley, UK) according to the manufacturer's protocol, although with minor modifications to ensure DNA‐yields and concentrations needed for subsequent analyses. Specifically, after adding Buffer AL, samples were incubated at 57°C, for 30 min, with 20 sec vortex every 10 min to ensure complete dissolution of the samples. In the final step only 100 *μ*L of the AE elution buffer was added to increase final concentration. The extraction quality of all samples was verified by the presence of high molecular weight bands on a 2% agarose gel. The DNA concentration was measured for all extractions with Qubit 2.0 (Life Technologies, Gaithersburg, MD).

### Genotyping

Population genomic data were generated using the Genotyping‐by‐Sequencing (GBS) approach (Elshire et al. 2011) – a method that is both economical and provides a relatively high output of single nucleotide polymorphisms (SNPs) distributed across the genome. Extracted DNA was processed by the GBS service provided by Cornell University's Institute of Biotechnology following their standard pipeline (Elshire et al. 2011). Initial sample optimization indicated the six base cutter restriction enzyme EcoT22I (target site ATGCA|T) exhibited effective genome fragmentation, and this was used for the GBS library generation. All samples were sequenced on Illumina HiSeq 2000 technology, using single read 64 bp chemistry (including library barcodes). Raw data are available from NCBI, accession SRP067014.

Initial data analysis used the zebra fish *Danio rerio*, Cyprinidae genome (NCBI assembly number GRCz10) as reference for the Tassel 4.3 pipeline (Bradbury et al. 2007). However, as only ca. 4% of the reads mapped, this approach was abandoned in favor of the UNEAK3 pipeline (Lu et al. 2013). Tags were defined as groups of more than five identical reads in the UMergeTaxaTagCountPlugin. To ensure a minimal amount of false SNPs to be included in the dataset, we set an Error Tolerance Rate (ETR) of 0.01 on the UTagCountToTagPairPlugin, and a minimum minor allele (MAF) frequency of 0.02 on the UMapInfoToHapMapPlugin. For all downstream analyses the SNPs with more than two alleles were removed. Finally, using Plink 1.9 (Purcell et al. 2007) all SNPs with more than 5% missing data were removed.

### Summary statistics and genomic population structure

GenoDive 2.0 (Meirmans and Van Tienderen 2004) was used to calculate summary statistics, including the observed frequency of heterozygotes within sampling sites (H_O_), the expected frequency of heterozygotes within sites (H_S_), also known as gene diversity, and the expected frequency of heterozygotes over all populations (H_T_). Other general statistics included were number of alleles, number of effective alleles, fixation index (*F*_ST_) and deviations from Hardy‐Weinberg equilibrium described as inbreeding coefficients (G_IS_) (10,000 permutations), with positive results meaning heterozygote deficiency, and negative results meaning excess of heterozygotes. Isolation by distance was assessed for *F*_ST_ values and Euclidean geographic distances, as well as *F*_ST_ values and waterway distances (Table S1) for all sample sites, using the Isolation‐By‐Distance Web Service (IBDWS) (Jensen et al. 2005). The IBD analyses were undertaken for both the full dataset and a dataset excluding the geographic distant KRO sample site.

Admixture version 1.23 was used to estimate ancestral relations of the sample sites (Alexander et al. 2009). The analysis was performed for 2–14 clusters, using default settings, and convergence was assessed by running the algorithm until the log‐likelihood difference between iterations was less than 10^−4^. Admixture output was plotted using an in‐house script, available from the authors upon request. The ancestral fractions for the most likely number of clusters, according to the Cross Validation (CV) error, were summed up for each sample site and plotted on a geographic map (Fig. 2) using ArcMap 10.3. The map also includes mean salinity data from the Baltic Sea (1999–2009) and North Sea mean salinity (2007–2008) (www.myocean.eu). Also displayed on the map was 10 × 10 km squares from which ide have been observed since 1995. This data were provided by the Natural History Museum of Denmark's extensive national fish atlas database (Carl & Møller, unpublished data). Also included were coastal areas of Sweden where ide have been reported (Kullander et al. 2012). In order to assess the connectivity of the samples and sample sites, a principal component analysis of the SNP dataset was undertaken using the SmartPCA software of the Eigensoft package (Patterson et al. 2006). The dataset were reduced to 10 eigenvectors, and vectors 1 and 2, and 1 and 3 were plotted using the Perl script Ploteig also included in the Eigensoft package.

## Results

### Extractions, sequencing, and filtering

DNA extractions yielded high molecular DNA, as envisioned by TA gel staining of bands >10,000 bp, and concentrations between 16.5 and 568 *μ*g/mL (Average = 157.4 *μ*g/mL; SD = 114.4). Large differences were observed in DNA yield between sample sites with POL, TRY and KOG having no samples reaching the desired 125 *μ*g/mL concentration whereas KRO had no samples with less than 125 *μ*g/mL of DNA (Fig. S1A).

Between 1,286,964 and 3,060,226 reads were produced for each ide GBS library (average of 2,105,232). Of these, sequences containing tags ranged from 234,277 to 430,808 (average of 322,466) (Fig. S1B). Sequencing depth (reads/tag) per SNP ranged from 5.49 to 7.10, with an average of 6.50 (Fig. S1D). Mean number of reads, mean number of tags and mean reads/tag in the sample sites did not seem to be affected by the varying DNA concentrations in the extracts (Fig. S1).

Data filtering demonstrated that prior to filtering the total dataset encompassed 40,429 SNPs, with all individuals exhibiting 32.5–45.0% missing data. It also revealed a linear relation between allowed missing data for each SNP and the number of SNPs kept in the dataset (*R* ^2^ = 0.991). The final dataset allowing only 5% missing data had 12,359 SNPs. In the filtered dataset, each individual had missing data in 64 (0.52%) to 534 (4.32%) SNPs.

### Summary statistics

The effective number of alleles in the total dataset was 1.319 (Table 1) and the effective numbers of alleles at the sample sites were between 1.309 and 1.398, with VID and TRY having the lowest and highest number respectively. The total expected heterozygosity (H_T_) level was 0.231 and for the sample sites the expected heterozygosity (H_S_) (gene diversity) ranged from 0.197 in VID to 0.259 in TRY. The observed heterozygosity (H_O_) for the sample sites was between 0.197 (VID) and 0.259 (TRY). Inbreeding coefficient (G_IS_) of the total dataset was −0.002, but not statistically significant. The inbreeding coefficients for the sample sites were between −0.042 and 0.033, but only the positive values were statistically significant, with localities KRO, OND, SUS, KOG, and LOD showing a deficit of heterozygotes. For the individual sample sites the inbreeding coefficients (G_IS_) were significant (*P* < 0.05) for 4.4–8.5% of SNPs within the sample sites, with an average of 6.1% (data not shown).

### Genomic population structure in relation to anadromous behavior

F_ST_ values ranged from 0.001 to 0.289, with the lowest pairwise values being between KOG and TRY sample sites, and the highest being between sample sites POL and VID (Table 2). The anadromous populations TRY, KOG, SUS, and LOD had lower *F*_ST_ values (from 0.001 to 0.047; 95% CI: 0.013–0.047) than freshwater resident populations, all of which had *F*_ST_ values between 0.135 and 0.289 (95% CI: 0.178–0.254), except for the KRO‐GUD comparison that had an *F*_ST_ of 0.070. All pairwise *F*_ST_ comparisons among sites were significantly different (*P* > 0.001), except KOG and TRY that had a very low *F*_ST_ value of 0.001 (*P* = 0.192).

**Table 2:** Population genetic differentiation of ide in north‐western Europe

	KRO	VID	GUD	OND	POL	SUS	TRY	KOG	LOD
KRO	–	0.001	0.001	0.001	0.001	0.001	0.001	0.001	0.001
VID	0.135	–	0.001	0.001	0.001	0.001	0.001	0.001	0.001
GUD	0.070	0.169	–	0.001	0.001	0.001	0.001	0.001	0.001
OND	0.183	0.262	0.209	–	0.001	0.001	0.001	0.001	0.001
POL	0.205	0.289	0.237	0.255	–	0.001	0.001	0.001	0.001
SUS	0.114	0.197	0.135	0.164	0.142	–	0.001	0.001	0.001
TRY	0.108	0.189	0.130	0.157	0.144	0.040	–	**0.192**	0.001
KOG	0.104	0.184	0.126	0.155	0.14	0.034	**0.001**	–	0.001
LOD	0.108	0.188	0.131	0.160	0.133	0.047	0.031	0.027	–

Pairwise *F*_ST_ values (bottom) and *P* values (top). Statistically insignificant comparisons are in bold.

**Figure 3:**
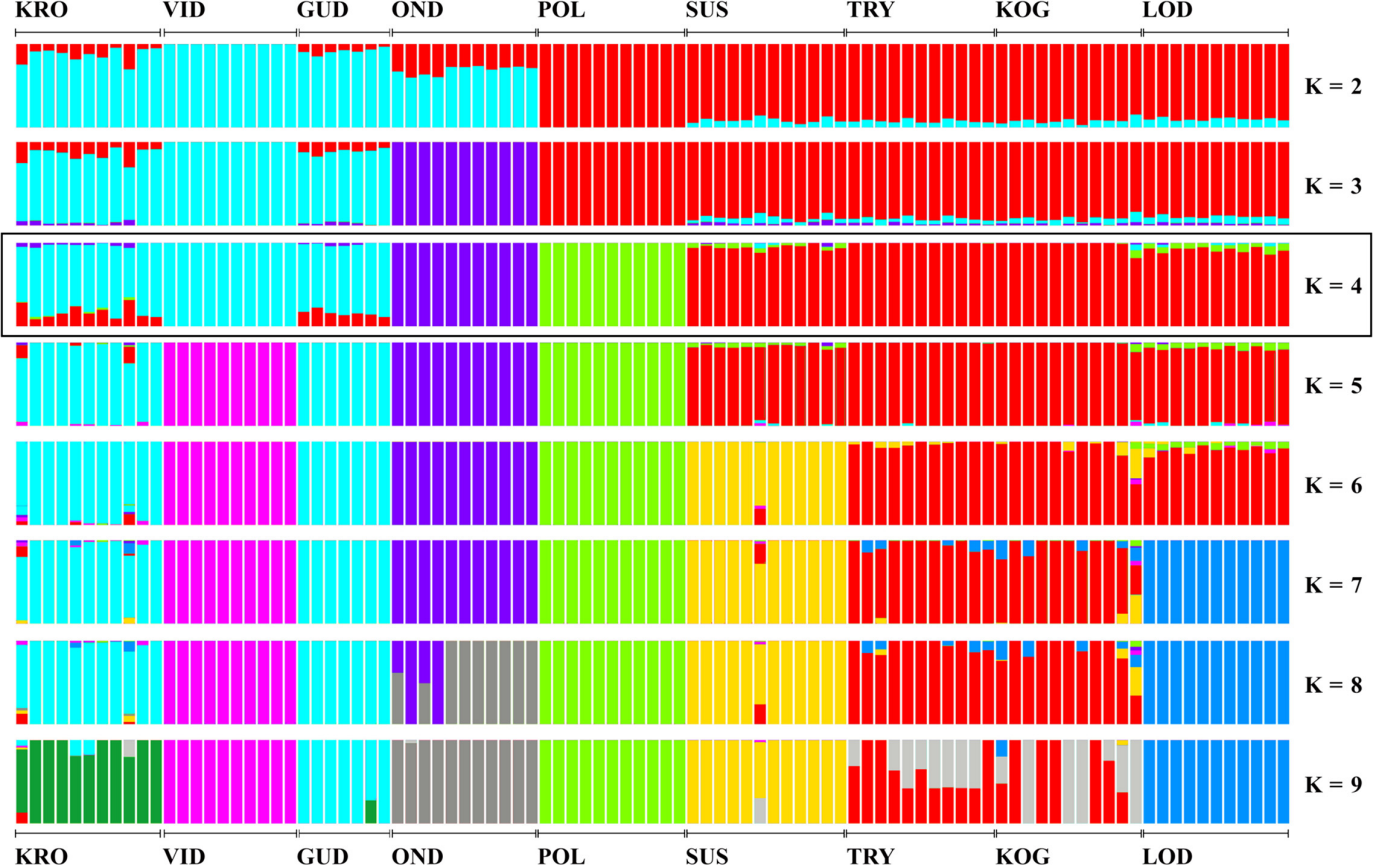
Plot of ancestral fractions from the Admixture cluster analysis. Each vertical bar represents one individual and the colours indicate the likelihood of it belonging to a particular ancestral population under the assumption that there were a given number (*K*) of such ancestral populations. Black square indicates the most likely number of clusters.

The cluster analysis implemented in Admixture showed that all individuals sampled from the same site have similar ancestral fractions (Fig. 3). The plot of two ancestral populations (*K* = 2) shows that there was a basic east‐west split between either sides of the marine channel known as ‘The Great Belt’ (Storebælt), that separates the two principal Danish islands of Funen (Fyn) and Zealand (Sjælland) (Fig. 2). When plotting three ancestral populations (*K* = 3), OND is the first site to separate from the other western sample sites. The most likely number of ancestral population was four (*K* = 4) (CV error rate = 0.48697) (Fig. S3) with the sample sites divided into (1) a western cluster consisting of KRO in the Netherlands and the two populations VID and GUD on the Jutland peninsula; (2) a cluster consisting of the OND sample site; (3) a cluster consisting of the POL site; and (4) the four eastern low‐salinity sample sites SUS, KOG, TRY, and LOD all clustering together (Fig. 2). In the western cluster, VID separates in the plot of five ancestral populations (*K* = 5) and KRO and GUD stay clustered together. This was consistent throughout plots for 5–8 ancestral populations despite VID being geographically in between KRO and GUD. In the eastern cluster sample sites SUS and LOD separate from the other eastern sample sites (KOG and TRY) in the plots for 6 (*K* = 6) and 7 (*K* = 7) ancestral populations respectively. Sample sites KOG and TRY, which are 3 km apart, cluster together for *K* = 2 through *K* = 8.

**Figure 4:**
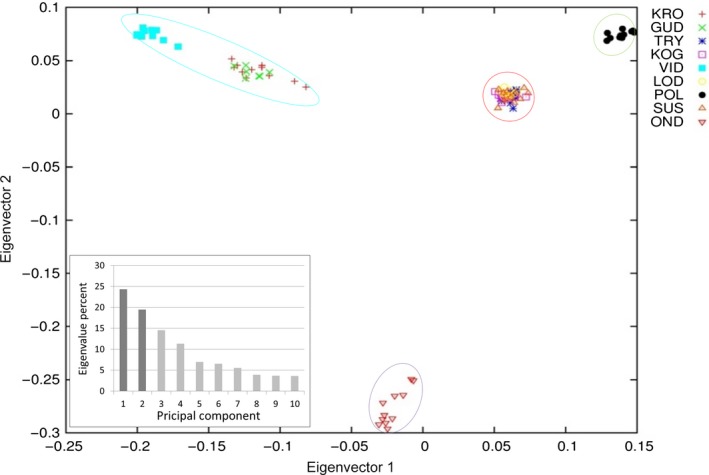
PCA plot of genetic variation in ide in north‐western Europe. Plot of eigenvector 1 and 2. Circles indicate the four most likely clusters in the Admixture analysis. Circle colors are uniform with those of four clusters in Fig. 3. Insert shows eigenvalue percent for each of the 10 eigenvectors. Darker columns indicate eigenvectors plotted.

**Figure 5:**
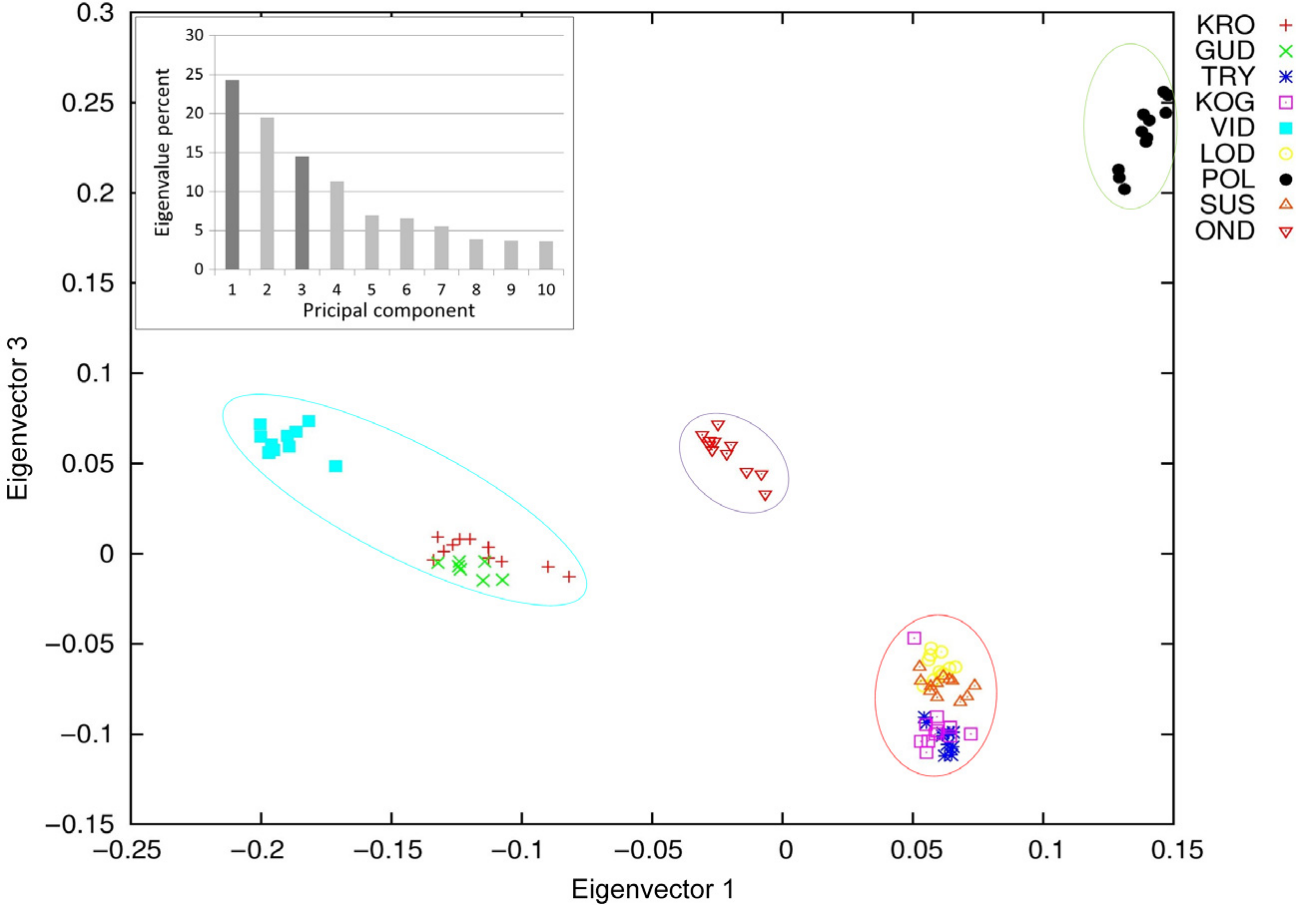
PCA plot of genetic variation in ide in north‐western Europe. Plot of eigenvector 1 and 3. Circles indicate the four most likely clusters in the Admixture analysis. Circle colors are uniform with those of four clusters in Fig. 3. Insert shows eigenvalue percent for each of the 10 eigenvectors. Darker columns indicate eigenvectors plotted.

The plot of eigenvectors show that the eastern sample sites LOD, SUS, KOG, and TRY cluster on top of each other (Figs. 4, 5). Sample sites GUD and KRO also cluster close to, but distinct from VID. Individuals from OND and POL form their own clusters. Eigenvector 2 shows a very pronounced separation of OND from all other sample sites (Figs. 4, 5) while eigenvector 3 shows an especially large separation of POL (Fig. 5). The eigenvalues show that 58 percent of the variation was found in eigenvectors 1, 2, and 3 combined.

Isolation by distance was statistically insignificant for the correlation of *F*_ST_ with both waterway distances (*P* = 0.364, *R* ^2^ = 0.036) and Euclidean geographic distances (*P* = 0.376, *R* ^2^ = 0.008) (Fig. S2). However, both the correlation of genetic similarity with waterway distances (*P* = 0.003, *R* ^2^ = 0.352) and Euclidean geographic distances (*P* = 0.006, *R* ^2^ = 0.397) were significant when not including the KRO sample site.

## Discussion

### Population history, salinity, and anadromous behavior

The Isolation by Distance (IBD) results reveal that geographic distance cannot explain all the genetic variation among sample sites. Rather, as suggested in studies of other freshwater species (e.g., perch *Perca fluviatilis*, spined loach *Cobitis taenia,* and bullhead *Cottus*) (Nesbö et al. 1999; Kontula and Väinölä 2001; Culling et al. 2006), the large scale patterns of population genomic structuring likely resulted from postglacial population histories and migration routes. Indeed, we observed a split between a western and an eastern clade, which likely reflect such major groups with different evolutionary histories.

At the finer micro‐evolutionary scale, all analyses consistently grouped the samples into four geographic units comprised of (1) LOD, KOG, TRY, and SUS; (2) KRO, VID, and GUD; (3) OND; and (4) POL. Anadromous ide populations are also characterized by lower levels of genetic differentiation than freshwater resident ones. This observation is in accordance with previous published comparisons of genetic differentiation between anadromous and freshwater species (Gyllensten 1985; Ward et al. 1994; DeWoody and Avise 2000), and is similar to the overall genomic population structure of other species with both anadromous and freshwater resident populations (e.g., Atlantic salmon *Salmo salar*) (Tonteri et al. 2007) and species with both brackish water and freshwater resident populations (e.g., zander *Sander lucioperca*) (Säisä et al. 2010). The level of genetic diversity within the anadromous group is also similar to that of other species (perch *Perca fluviatilis* and whitefish *Coregonus maraena*) with possible interpopulation gene flow in the Baltic Sea (Olsson et al. 2011, 2012). Given this, we hypothesize that one factor affecting the observed genomic population structure of ide is gene flow between anadromous populations in regions with salinities within the tolerance of ide, and reduction or cessation of gene flow in regions with salinities above the ide's salinity tolerance. This hypothesis is further supported by observations of ide in marine habitats occurring in these regions (Fig. 1,2).

Although the salinity levels allow for frequent gene flow among the four anadromous sampling localities, our results suggest some degree of population genomic structure among these sites. In line with previous observations of freshwater resident ide populations (Winter and Fredrich 2003), as well as anadromous fish species in general (McDowall 2001), we suggest that this fine‐scale genetic structuring derives from an affinity of individuals to spawn at their natal site (i.e., homing). Our observation that the geographically close TRY and KOG streams form a single population may result from a lack of analytical power to differentiate them, or from the fishes’ inability to distinguish between geographically close streams, as has been reported for anadromous species such as the alewife *Alosa pseudoharengus* and blueback herring *Alosa aestivalis* (Palkovacs et al. 2014).

In the future, when ocean water levels rise and the salinities change in river deltas around the world, anadromous populations will be facing new challenges. For well‐studied, salinity tolerant, anadromous species the focus has been on temperature (Reist et al. 2006; Jonsson and Jonsson 2009; Hedger et al. 2013), but for anadromous populations of true freshwater fishes that exhibit a wider temperature range, increased salinity could play a more important role. For the populations of anadromous ide that are already close to their maximum salinity tolerance, our data indicate that potential increases in salinity will almost certainly drive populations to isolation, as seen in the current freshwater populations. As a result, we predict increased genetic differentiation between populations, decreased genetic diversity, and the restriction of anadromous behavior to regions in the eastern Baltic Sea with lower and more stable salinity levels. In contrast, should salinity in the western Baltic Sea decrease, the effects will be increased gene flow, which could lead to loss of local adaptive traits, or alternatively, the genetic “rescue” of isolated populations.

### Local genomic population structure and possible effects of human impact?

In the cluster that includes all the anadromous populations, we note that the TRY and KOG populations are more closely connected to the Swedish sample site LOD, located 55 km away on the other side of the high‐current Øresund strait, than they are to the SUS sample site located 120 km along the coast. If this is a result of modern events it could suggest that ide migrate not only along the coast‐line, but also cross deeper waters, and indicate that the waterway distance might play an important role in limiting gene flow. However, this cannot be determined in this study, thus future telemetric studies of ide in this region would represent a means to shed light on such behavior. The freshwater resident population POL is closely related to the anadromous sample sites TRY, KOG, LOD, and SUS. This close relationship could be explained by joined postglacial history or maybe POL could historically have been anadromous with low levels of gene flow, as Lake Arresø was a fjord until the 17th century (Naturstyrelsen 2015).

Sample sites GUD, KRO, and VID are all located on the European mainland, and contain ide belonging to a single genetic group. Among these, the similarity of the KRO and GUD sample sites is particularly noteworthy. The population present at the GUD site was first noticed in the 1970's, and its gradual movement downstream in the GUD system has since been reported by both local anglers and monitoring programs (Jensen 2006; Carl 2012a). Although these ide are believed to have originated from a single stocking event of 400–500 individuals, the origins of these fish are unknown. In this regard, it is possible that they were illegally imported from Germany, something that has been reported in this region for at least one species of reptile (Jensen 2002) and Wels catfish (*Silurus glanis*) (Carl 2012b). The KRO sample site is part of the Rhine water system that runs through Germany. Furthermore, a study from another central European river showed that ide living in a 120 km stretch is a “single panmictic unit” (Wolter et al. 2003), supporting the hypothesis that German ide might be closely related to ide from the KRO sample site in the Netherlands. Further support for a hypothesis of ide introduction from the Rhine into GUD comes from the observation that F_ST_ value of the comparison between GUD and KRO ide is lower than that for other landlocked sample sites. On the other hand, one would expect a lower level of heterozygosity as a consequence of a recent founder effect (Nei et al. 1975; Wright 1990), but we do not see this for GUD ide. However, if the GUD ide consist of a mix of original and introduced specimens, genetic diversity may be higher than expected. Therefore, the true origin of this population will need to be further studied before any firm conclusions can be made.

A last point of note relating to the ide in this study, are those from the Odense Å (OND) stream. Archaeological excavations have indicated that this population has been present since at least the Iron Age (Gotfredsen et al. 2009), and until the 1930's they were so abundant that they were used for fertilizing the local fields (Frederiksen 1979). However, during industrialization of the region between the 1940's and 1980's the river was used as an open sewer, leading to dramatic declines in the ide population (Carl 2012a). Given this, we note that our observation of a genetically unique population there today, exhibiting variation at levels similar to other sample sites, suggests that the population nevertheless not only survived, but may have avoided a genetic bottleneck despite this pollution (Bickham et al. 2000).

In summary, our results provide a first look into the genomic population structure of ide in north‐western Europe, and lay the foundation for further studies. In this regard, telemetric studies of migration in anadromous populations, and the search for genetic markers linked to salinity adaptation, will be of considerable interest. Another research field that could build upon the present study is analyses of postglacial colonization and demographic history. The ide is a species well suited for such studies as it has not been systematically stocked and redistributed by man. During the last decade we have seen an increased interest in the ide from recreational anglers practicing catch‐and‐release. This may lead to a change in status of the ide from nuisance fish and agricultural fertilizer, to an appreciated recreational species and maybe even future introduction of management strategies.

## Conflict of Interest

None declared.

## Supporting information


**Figure S1.** Mean DNA concentrations, mean reads, mean tags and mean reads/tag for all sample sites.

**Figure S2.** Isolation by distance in ide in north‐western Europe.

**Figure S3.** Cross Validation (CV) error rate of admixture analysis of ide.

**Table S1.** Geographic distance (km) between ide *Leuciscus idus* sample sites. Euclidean distance (top) and waterway distance (below).
