# Erratum

**DOI:** 10.1002/ece3.2086

**Published:** 2016-03-17

Mikkel Skovrind, Morten Tange Olsen, Filipe Garrett Vieira, George Pacheco, Henrik Carl, M. Thomas P. Gilbert, Peter Rask Møller. Genomic population structure of freshwater‐resident and anadromous ide (*Leuciscus idus*) in north‐western Europe. Ecology and Evolution 2016; 6, 1064–1074 DOI:10.1002/ece3.1909

In the above‐mentioned article, there are three typos where “%” should have been “‰”. The typos were introduced during the proof process and were not in the manuscript presented to the reviewers. We apologize for the errors.

The fourth sentence of the abstract should say: “The idea is a stenohaline freshwater fish that primarily inhabits rivers, with frequent anadromous behavior when sea salinity does not exceed 15‰.”

The fourth sentence of the introduction should say: “The Baltic Sea is a mega estuary with salinities ranging from 0 to 20‰ (Janssen et al. 1999)…”

The first sentence of the second paragraph in the introduction should say: “The maximum tolerated salinity for ide (Fig. 1) is 15‰ (Penthon 1985; van Beek 1999).”

The corrections made to this article are not of a nature that affects the results nor the interpretation included in the study.

