# Supplementary figures and images for: Genomic population structure of freshwater‐resident and anadromous ide (Leuciscus idus) in north‐western Europe
Source: Ecol Evol. 2016 Jan 22;6(4):1064–74. doi: 10.1002/ece3.1909 (PMC4761760; doi:10.1002/ece3.1909)

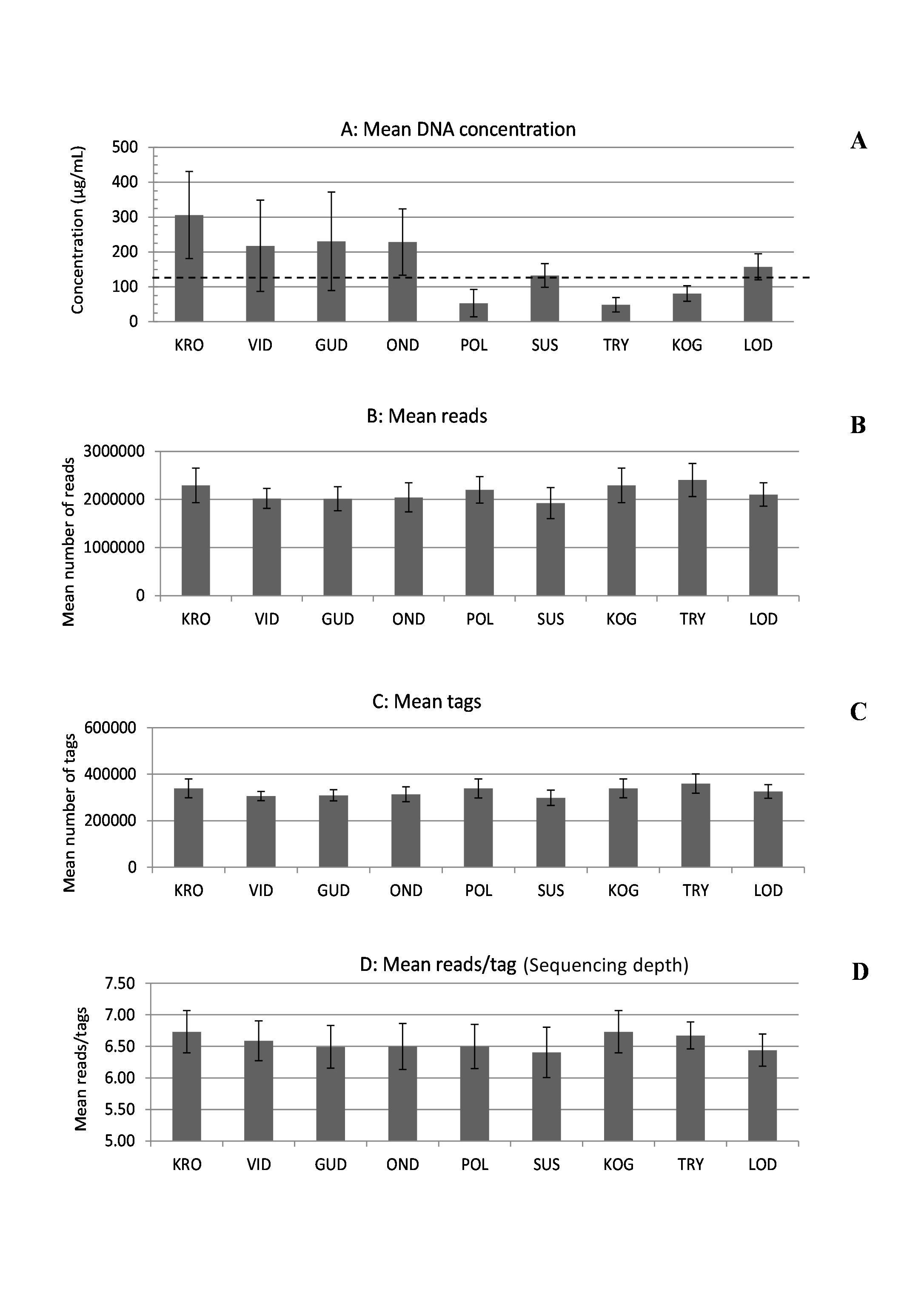

Supplement: Supplementary file 1 — Figure S1. Mean DNA concentrations, mean reads, mean tags and mean reads/tag for all sample sites. [file ECE3-6-1064-s001.tif]

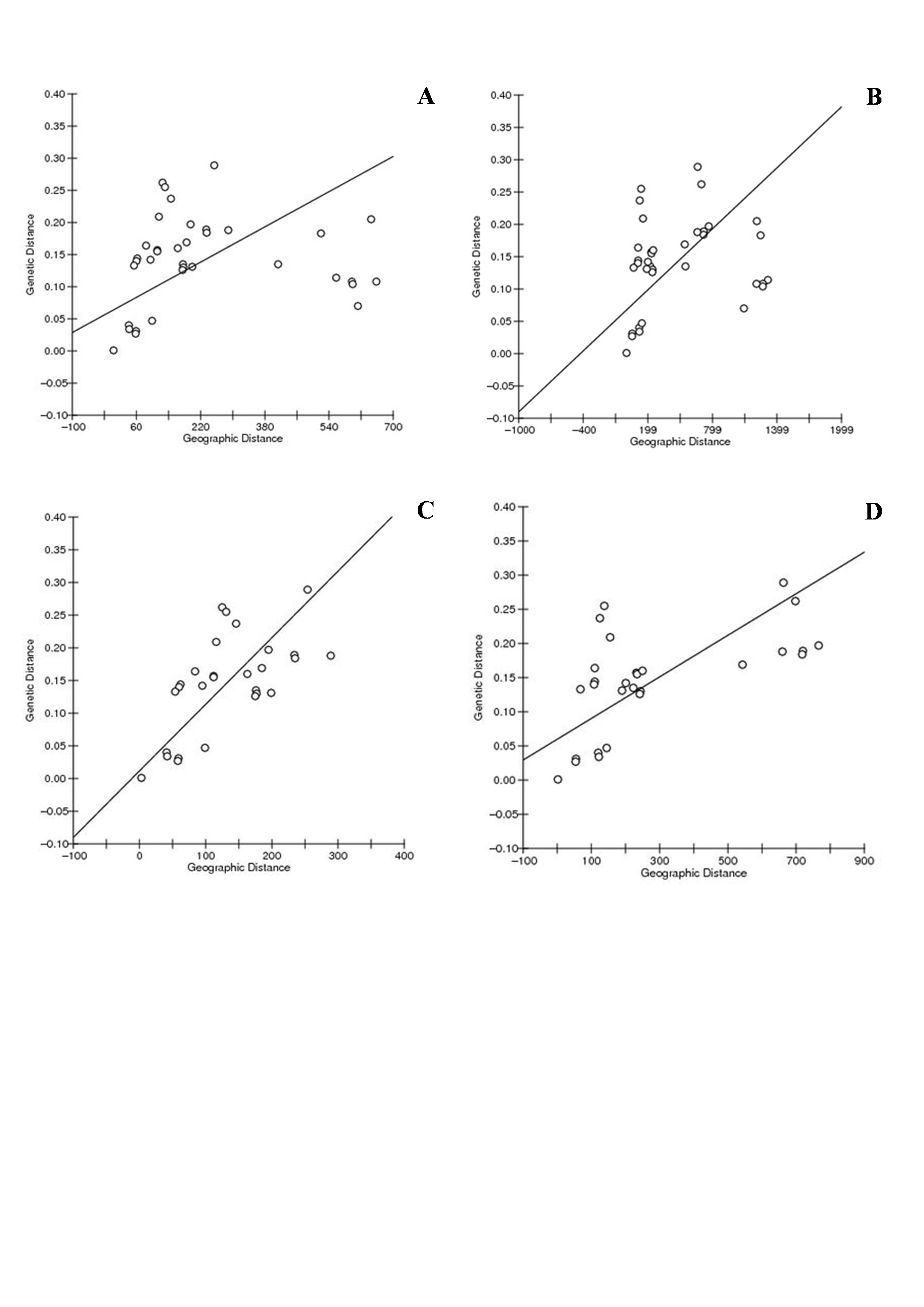

Supplement: Supplementary file 2 — Figure S2. Isolation by distance in ide in north‐western Europe. [file ECE3-6-1064-s002.tif]

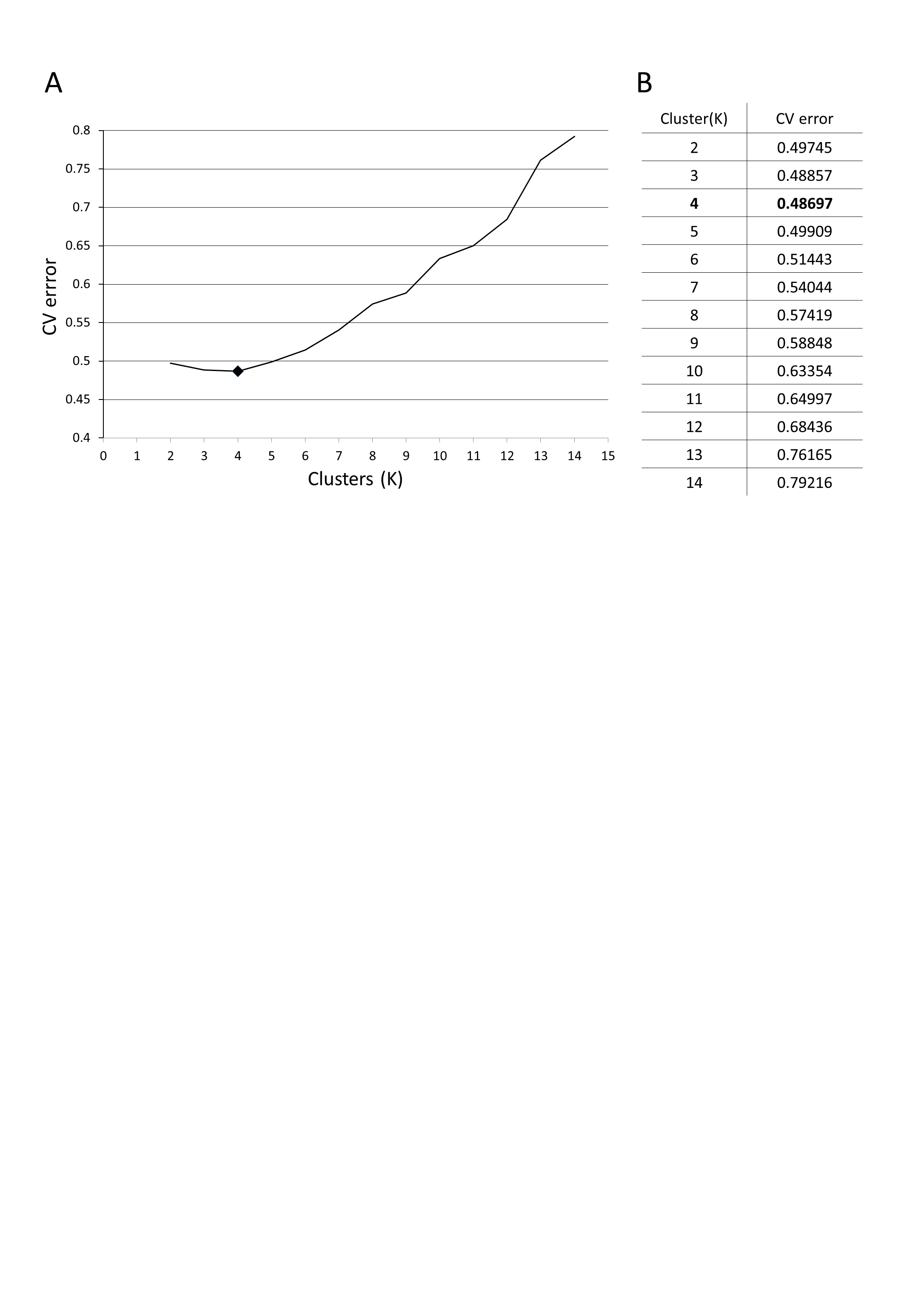

Supplement: Supplementary file 3 — Figure S3. Cross Validation (CV) error rate of admixture analysis of ide. [file ECE3-6-1064-s003.tif]
