# Supplementary material for: Genomic population structure of freshwater‐resident and anadromous ide (Leuciscus idus) in north‐western Europe
Source: Ecol Evol. 2016 Jan 22;6(4):1064–74. doi: 10.1002/ece3.1909 (PMC4761760; doi:10.1002/ece3.1909)
Supplement: Supplementary file 4 — Table S1. Geographic distance (km) between ide Leuciscus idus sample sites. Euclidean distance (top) and waterway distance (below). [file ECE3-6-1064-s004.docx]

| Table S1. Geographic distance (km) between ide *Leuciscus idus* sample sites. Euclidean distance (top) and waterway distance (below). | | | | | | | | | |
| --- | --- | --- | --- | --- | --- | --- | --- | --- | --- |
|  | KRO | VID | GUD | OND | POL | SUS | TRY | KOG | LOD |
| KRO | -- | 413 | 612 | 520 | 645 | 558 | 597 | 599 | 658 |
| VID | 550 | -- | 185 | 125 | 254 | 195 | 234 | 235 | 289 |
| GUD | 1093 | 543 | -- | 116 | 146 | 176 | 177 | 175 | 199 |
| OND | 1248 | 698 | 155 | -- | 131 | 84 | 112 | 112 | 163 |
| POL | 1213 | 663 | 125 | 138 | -- | 95 | 62 | 60 | 54 |
| SUS | 1316 | 766 | 223 | 110 | 201 | -- | 41 | 42 | 99 |
| TRY | 1270 | 720 | 244 | 232 | 110 | 120 | -- | 3 | 59 |
| KOG | 1268 | 718 | 242 | 234 | 108 | 122 | 3 | -- | 58 |
| LOD | 1210 | 660 | 190 | 250 | 68 | 145 | 55 | 54 | -- |
